# The role of melatonin in plant growth and metabolism, and its interplay with nitric oxide and auxin in plants under different types of abiotic stress

**DOI:** 10.3389/fpls.2023.1108507

**Published:** 2023-02-14

**Authors:** Irshad Ahmad, Xudong Song, Muhi Eldeen Hussein Ibrahim, Yousaf Jamal, Muhammad Usama Younas, Guanglong Zhu, Guisheng Zhou, Adam Yousif Adam Ali

**Affiliations:** ^1^ Joint International Research Laboratory of Agriculture and Agri-Product Safety of the Ministry of Education of China, College of Agriculture, Yangzhou University, Yangzhou, China; ^2^ Department of Agronomy, Institute of Agricultural, Sudan University of Science and Technology, Khartoum, Sudan; ^3^ Jiangsu Yanjiang Area, Institute of Agricultural Sciences, Nantong, China; ^4^ Department of Agronomy, Faculty of Agriculture, University of Swabi, Swabi, Pakistan; ^5^ Department of Crop Genetics and Breeding, College of Agriculture, Yangzhou University, Yangzhou, China; ^6^ Key Lab of Crop Genetics & Physiology of Jiangsu Province, Yangzhou University, Yangzhou, China; ^7^ Department of Agronomy, Faculty of Agricultural and Environmental Science, University of Gadarif, Al Gadarif, Sudan

**Keywords:** abiotic stresses, auxin, nitric oxide, phytomelatonin, plant growth and metabolism

## Abstract

Melatonin is a pleiotropic signaling molecule that reduces the adverse effects of abiotic stresses, and enhances the growth and physiological function of many plant species. Several recent studies have demonstrated the pivotal role of melatonin in plant functions, specifically its regulation of crop growth and yield. However, a comprehensive understanding of melatonin, which regulates crop growth and yield under abiotic stress conditions, is not yet available. This review focuses on the progress of research on the biosynthesis, distribution, and metabolism of melatonin, and its multiple complex functions in plants and its role in the mechanisms of metabolism regulation in plants grown under abiotic stresses. In this review, we focused on the pivotal role of melatonin in the enhancement of plant growth and regulation of crop yield, and elucidated its interactions with nitric oxide (NO) and auxin (IAA, indole-3-acetic acid) when plants are grown under various abiotic stresses. The present review revealed that the endogenousapplication of melatonin to plants, and its interactions with NO and IAA, enhanced plant growth and yield under various abiotic stresses. The interaction of melatonin with NO regulated plant morphophysiological and biochemical activities, mediated by the G protein-coupled receptor and synthesis genes. The interaction of melatonin with IAA enhanced plant growth and physiological function by increasing the levels of IAA, synthesis, and polar transport. Our aim was to provide a comprehensive review of the performance of melatonin under various abiotic stresses, and, therefore, further explicate the mechanisms that plant hormones use to regulate plant growth and yield under abiotic stresses.

## Introduction

Abiotic stresses continuously reduce the growth and yield of different crops (Zhang et al., 2021a; Ahmad et al., 2022a). The growing of plants in altered environments often creates abiotic stresses such as salinity, drought, heat, cold, and heavy metals. The imposition of abiotic stresses can certainly affect plants’ morphophysiological, biochemical, and molecular activity, from seed germination to maturity, and, eventually, cause higher losses in plant yields (Rahman et al., 2022). It has been demonstrated that about 70% of staple food crop yields are adversely affected by abiotic stresses (Khan et al., 2015). These stresses induce numerous changes in the metabolism of plants by producing reactive oxygen species (ROS), which in turn disturb homeostasis and ion distribution in plants (Raza et al., 2022). Improving the response of plants to these stresses is particularly important for sustainable plant production (Gonzalez Guzman et al., 2022). Over the last few decades, tremendous efforts have been made by research scientists to enhance plant growth and yields *via* the extensive application of chemicals.

Melatonin (*N*-acetyl-5-methoxytryptamine) is an important bioactive compound in vascular plants, discovered in 1995 (Dubbels et al., 1995). Initially, it was regarded as a powerful antioxidant that had different beneficial roles in various stages of plant growth and development (Sheshadri et al., 2018), such as germination (Zhang et al., 2017), root elongation (Arnao and Hernández-Ruiz, 2019), photosynthesis (Li et al., 2017), and leaf senescence (Wang et al., 2022). It has also been a plant hormonewith an important role in enhancing the growth and regulation of plants (Arnao and Hernández-Ruiz, 2019). It is found in various plants tissues, such as the seeds, roots, leaves, and fruits (Zhang et al., 2017). Thepotential role melatonin could play in the enhancement of plant growth andregulation has been widely investigated by scientific researchers (Sun et al., 2020).

Recently, it has been reported that melatonin increases the fatty acid content and enhances the profile of alkaloids in coffee and soybean plants (Ramakrishna et al., 2012). However, the mechanism of enhanced fatty acid production *via* melatonin is far from clear and needs to be further investigated in different crops under various abiotic stresses. As a multiregulatory molecule, melatonin regulates the expression of genes involved in plant growth and development (Byeon and Back, 2014), redox reactions (Tomas and Montes, 2005), abiotic stress resistance (Boccalandro et al., 2011), sucrose metabolism [cell wall invertase (CWIN) and sucrose synthase (SUSY)] (Solfanelli et al., 2006; Dutta et al., 2013; Payyavula et al., 2013), and specialized metabolism [phenylpropanoid metabolism: phenylalanine ammonia lyase (PAL), chalcone synthase (CHS), chalcone isomerase (CHI), flavanone 3-hydroxylase (F3H), dihydroflavonol reductase (DFR), and anthocyanidin synthase (ANS)] (Weeda et al., 2014). The phytomelatonin receptor PMTR1 mediates the signaling of ROS, regulates homeostasis or, and delivers a dark indication to promote night stomatal closure (thus avoiding water loss during the night), thereby facilitating plant adaptation to dry land environments (Li et al., 2020). However, what genes participate in the signaling pathway to promote night stomatal closure, and how these genes evolved to facilitate plant adaptation to dry land environments, is still far from clear. In addition, because of the limitation of experimental methods, there is still no definitive evidence showing that melatonin function in plant organs is significantly enhanced at night as compared with the daytime (Van Tassel et al., 2001; Xie et al., 2022). The findings of various studies related to the role of phytomelatonin are of huge significance (Zhang et al., 2021b). Melatonin gives plants resistance to drought (Wang et al., 2014), salt (Hernández et al., 2015), osmotic stress (Zhang et al., 2013), high temperature (Byeon and Back, 2014), cold (Bajwa et al., 2014), and copper stress (Posmyk et al., 2009a).

It has been confirmed that the application of exogenous melatonin can mitigate the effects of abiotic stresses in various crops (Cao et al., 2019). Lower doses of melatonin (i.e., <10 μM) have been shown to promote seed germination and lateral root formation in cucumber plants under cold and drought stresses (Zhang et al., 2013; Simlat et al., 2018). In corn seedlings, melatonin increased drought resistance by alleviating oxidative damage and drought-induced photosynthetic inhibition (Ye et al., 2016). The pretreatment of melatonin also increased endogenous melatonin and inhibited the up-regulation of *NCED1* genes, but selectively up-regulated catabolic genes, such as *ABA80x1* and *ABA80x3*, and abscisic acid (ABA)-related synthesis genes, and decreased the accumulation of ABA and induced stomatal reopening in corn under drought stress (Li et al., 2021). In apple trees, melatonin maintained drought tolerance by regulating the concentrations of ABA metabolism and stomatal behavior (Li et al., 2015). In barley, the exogenous supply of melatonin increased photosynthetic carbon assimilation by improving the antioxidant defense of organelles under low temperature or drought stresses (Li et al., 2016). To date, most of the components in melatonin-related signaling pathways remain unclear and need to be further investigated, especially in plants under abiotic stresses (Zhou et al., 2020). In previous studies, melatonin has been shown to be present at high concentrations in several crops (e.g., wheat, rice, barley, corn, grape, oats, and tobacco), and in popular beverages (e.g., tea, coffee, and wine) (Arnao and Hernández-Ruiz, 2009; Ramakrishna et al., 2012; Arnao and Hernández-Ruiz, 2013; Shi et al., 2015a). However, it is still unknown if the response of melatonin in plants under various stresses is the same across different crops.

Therefore, in this manuscript we have aimed to provide a comprehensive review of advances in our knowledge of the roles, biosynthesis, distribution, metabolism, functions, and mechanisms of melatonin in regulating the growth and development of various crops under abiotic stresses. In addition, the interactions of melatonin with other phytohormones, such as nitric oxide (NO) and auxin (IAA, indole-3-acetic acid), are analyzed.

## Melatonin biosynthesis

**Figure 1:**
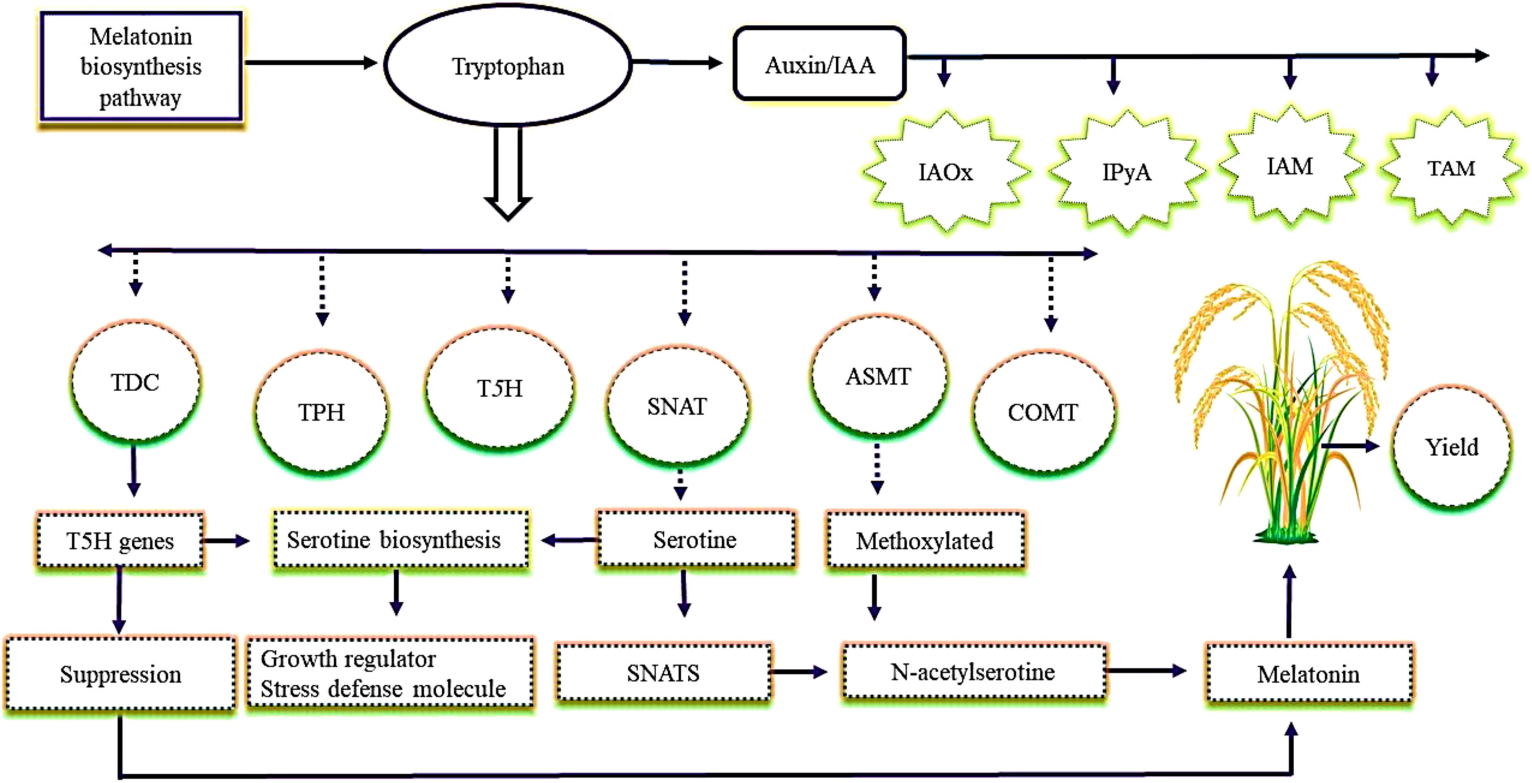
The regulatory role of biosynthetic melatonin under stress conditions. Tryptophan is the initial substrate of melatonin synthesis and is divided into four enzymatic steps catalyzed by six enzymes: tryptophan decarboxylase (TDC), tryptophan hydroxylase (TPH), tryptamine 5‐hydroxylase (T5H), serotonin *N*‐acetyltransferase (SNAT), *N*‐acetylserotonin methyltransferase (ASMT), and caffeic acid *O‐*methyl transferase (COMT). Serotonin is catalyzed *via* SNAT to form *N-*acetylserotonin, which is further methoxylated by ASMTs to form melatonin and acts as a growth regulator stress defense molecule. The *T5H* gene improves serotonin biosynthesis. TDC catalyzes decarboxylation of tryptophan, and it is considered the first step of melatonin biosynthesis. Suppression of the *T5H* gene increases the concentration of melatonin in rice plants and increases yield. Auxin produced naturally in plants is biosynthesized from tryptophan in four ways: indole-3-acetaldoxime (IAOx), indole-3-pyruvic acid (IPyA), indole-3-acetamide (IAM), and tryptamine (TAM).

The biosynthetic pathway of melatonin in plants is well documented (Park et al., 2012; Kang et al., 2013). The concept of plant-synthesized melatonin was first introduced in an isotope tracer study (Murch et al., 2000). The biosynthetic pathway of phytomelatonin in vascular plants is thought to be similar to that in animals, although there is much debate surrounding this (Murch et al., 2000; Tan et al., 2013; Zhao et al., 2019). Based on a number of findings, tryptophan is considered as the initial substrate of melatonin synthesis and is involved in four enzymatic steps catalyzed by at least six enzymes, including tryptophan decarboxylase (TDC), tryptophan hydroxylase (TPH), tryptamine 5‐hydroxylase (T5H), serotonin *N*‐acetyltransferase (SNAT), *N*‐acetylserotonin methyltransferase (ASMT), and caffeic acid *O‐*methyl transferase (COMT) (Back et al., 2016; Sun et al., 2021) (**Figure 1**). The two required processes that contribute to tryptophan are hydroxylation and decarboxylation for melatonin biosynthesis. They have been identified in herbivorous plants (Park et al., 2012). Auxin [indole-3-acetic acid (IAA)], which occurs naturally in plants, is biosynthesized from tryptophan *via* four proposed routes, that is, indole-3-acetaldoxime (IAOx), indole-3-pyruvic acid (IPyA), indole-3-acetamide (IAM), and tryptamine (TAM). The biosynthesis pathway of auxin from tryptophan is still unknown and needs to be further investigated in different crops under abiotic stresses. Serotonin is catalyzed *via* SNATs to form *N*-acetylserotonin, which is then methoxylated by ASMTs to form melatonin (Wang et al., 2017). Serotonin performs various important functions in plants, such as growth regulation and stress defense (**Figure 1**). Currently, the presence and function of serotonin in plants is an increasingly popular research area, but to date, there are only minor studies available about the functions of serotonin under different abiotic stresses. It has been shown that in rice TDC-catalyzed decarboxylation of tryptophan is the first step in melatonin biosynthesis, followed by T5H‐catalyzed hydroxylation (Park et al., 2012). The *T5H* gene is considered an essential gene for serotonin biosynthesis. It has been found that suppression of the *T5H* gene in transgenic rice increases the melatonin concentration, suggesting that melatonin concentration in plants is not proportional to serotonin concentration (Sun et al., 2021). The increase in melatonin concentration and the up-regulation of the *T5H* gene for serotonin biosynthesis under abiotic stresses in other crops are still far from being clearly understood.

## Role of melatonin in plants under abiotic stresses

### The distribution, metabolism, and complex functions of melatonin in plants under abiotic stresses

**Figure 2:**
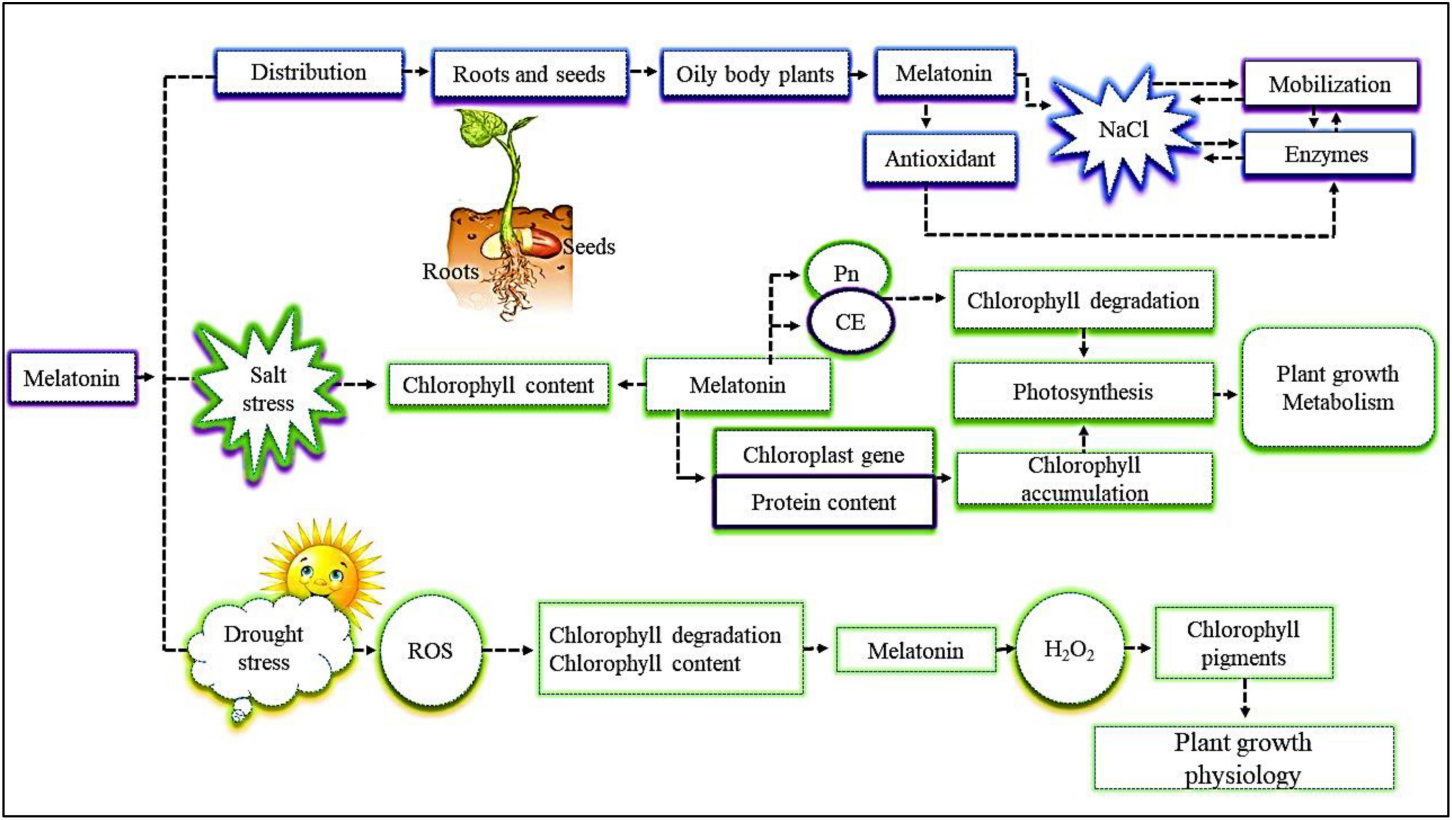
The distribution and regulatory roles of an exogenous supply of melatonin in mitigating abiotic stresses are divided into three parts. (i) Melatonin distributed in the roots and seeds. Sodium chloride (NaCl) induces slow mobilization of enzymes and reduces the enzymes’ activity in the oily body of plants in seedlings and roots and, as a result, alters salt stress. The supply of exogenous melatonin enhances the level of plant antioxidants and enzyme activities. (ii) Salt stress reduces chlorophyll content, but exogenous melatonin improves chlorophyll content, chloroplast gene expression, and protein content, and, as a result, enhances photosynthesis activity and chlorophyll accumulation. In addition, it enhances plant *Pn* and *CE* and, as a result, inhibits chlorophyll degradation. The improvement of all these traits enhances plant growth and metabolism. (iii) Drought stress causes ROS in plants, which induces *Ch* degradation and *Ch* reduction. The application of endogenous melatonin during drought reduces ROS and O_2_^–^ content and, as a result, increases chlorophyll content and plant growth physiological function. *CE*, carboxylation efficiency; *Ch*, chlorophyll; *Pn*, net photosynthetic rate; ROS, reactive oxygen species.

The immunohistochemical localization of melatonin has demonstrated that the compound is present in the primary roots and seeds of sunflower and *Arabidopsis* seedlings (Pelagio-Flores et al., 2012; Mukherjee et al., 2014; **Figure 2**). The accumulation of melatonin was observed in the oily body of plants, including in the cotyledon cells of both control and salt-treated seedlings, thus showing the effect of long-distance signaling, induced by sodium chloride (NaCl) stress, from roots to cotyledons (Mukherjee et al., 2014). A study found that NaCl stress induced slower mobilization in the cotyledons of sunflower seedlings (David et al., 2010). NaCl stress caused melatonin accumulation in seedling cotyledons, and, as a result, reduced degradation of the oily body. The mobilization of the oily body and the activity of fatty acid-metabolizing enzymes are considered to mitigate the effects of salt stress (David et al., 2010). The accumulation of melatonin in cotyledons played a positive antioxidative role, in that it maintained the activity of the enzymes required for lipid mobilization during seedling growth (David et al., 2010). The accumulation mechanism of melatonin in the oily body of plants, including in the cotyledon cells of control and salt-treated seedlings, is less well documented, and further studies are required so that, ultimately, antioxidant defense systems of various crops can be improved.

Moreover, melatonin can trigger the accumulation of nitric oxide *via* its up-regulation of nitrate reductase expression and down-regulation of *S*-nitrosoglutathione reductase (GSNOR) expression. The application of melatonin can alter the levels of NO in plants, and, as a result, affect the level of endogenous melatonin. The molecular interaction mechanisms of melatonin and NO are indispensable to different physiological activitiesin plants. However, the molecular interaction mechanisms of melatonin with NO in plants is still far from clear (He and He, 2020). Melatonin can mediate the crosstalk between NO and ethylene and regulate the ripening of fruits *via N*-nitrosomelatonin (NOMela) signaling (Mukherjee, 2019). In pear fruits, for example, melatonin reduced ethylene production and delayed post-harvest senescence by regulating NO synthesis (Liu et al., 2019). A recent discovery identified that the interaction between melatonin and NO resulted in the formation of NOMela (i.e., *N*-nitrosomelatonin), and the promised roles in plant morphophysiological activity (Martínez-Lorente et al., 2022). However, owing to the limited available knowledge on melatonin, the interaction of these two compounds and fruit ripening occurring *via* NOMela in various plants are poorly understood when these plants are under abiotic stresses. In addition, melatonin induces the accumulation of IAA *via* NO and, as a result, affects the formation of adventitious roots in tomato seedlings (Xie et al., 2022). Melatonin also regulates the transport and distribution of auxin, in turn promoting the formation of adventitious roots in tomato plants (Wen et al., 2016).

Melatonin has been widely shown to promote plant growth and photosynthetic activity under salt stress (Wang et al., 2016; **Figure 2**). Melatonin greatly reduced the decrease in chlorophyll *a* (*Chl a*), chlorophyll *b* (*Chl b*), and total chlorophyll (*Chls*) contents caused by salt stress, and promoted the net photosynthetic rate (*Pn*) and carboxylation efficiency (*CE*), showing that it can alleviate chlorophyll degradation caused by salt stress in plants (Kudoh and Sonoike, 2002; Yin et al., 2019). Previous studies found that the contents of *Chl a*, *Chl b*, and *Chls* were much higher in plants treated with melatonin than in untreated plants, indicating that melatonin facilitated both chloroplast gene expression and protein content turnover to promote the accumulation of chlorophyll content (Suo et al., 2015; Siddiqui et al., 2019). Melatonin can act as an antioxidant agent, reducing ROS activity and, as a result, inhibits chlorophyll degradation (Ma et al., 2018; **Figure 2**). There are studies that indicate that melatonin reduces the degradation of chlorophyll by down-regulating the expression of chlorophyll degradation-related genes during methyl jasmonate-induced senescence (Wang et al., 2019). However, more studies are needed to identify the various genes and measure gene expression involved in reducing chlorophyll degradation in various plants under abiotic stresses.

### Interaction of melatonin with nitric oxide

**Figure 3:**
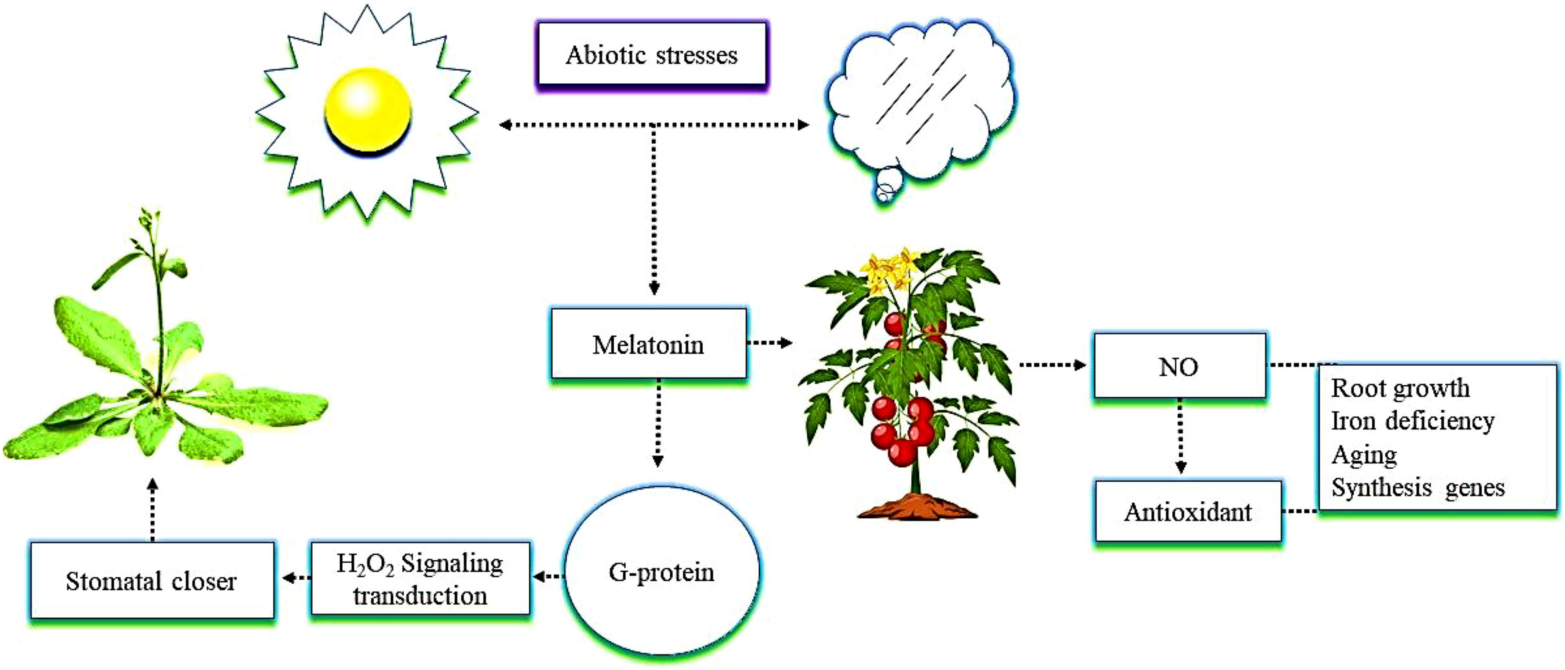
The interactive role of melatonin with nitric oxide (NO) in mitigating abiotic stress. The application of melatonin in tomato plants enhances NO content. NO further triggers antioxidant enzymes activity in plants, which shows resistance to abiotic stress and enhances root growth, iron deficiency, aging, and the expression of synthesis genes. In addition, G protein as a melatonin receptor enhances root growth, iron deficiency, aging, and the expression of synthesis genes mediates hydrogen peroxide (H_2_O_2_) signaling transduction that is involved in a melatonin-induced stomatal closure in *Arabidopsis*.

It is essential to study the physiological responses of crops regulated by the interactions between melatonin and NO to ensure higher yields of these crops. Melatonin and NO affect several physiological processes, such as root growth, mitigation of iron deficiency, and aging (Kaya et al., 2020; **Figure 3**). The interactions between the two compounds regulate many genes involved in hormone synthesis and, as a result, change the levels of phytohormones (Zhu et al., 2019; Singhal et al., 2021). Interactions between melatonin and NO have recently been identified under conditions of plant stress (Arnao and Hernández-Ruiz, 2018). A previous study demonstrated that melatonin triggers the endogenous accumulation and synthesis of NO, which acts as an antioxidant and regulates other plant defense mechanisms (Okant and Kaya, 2019). The G protein-coupled receptor, as a melatonin receptor, mediates hydrogen peroxide (H_2_O_2_) signaling transduction, which is in turn involved in melatonin-induced stomatal closure in *Arabidopsis* plants (Wei et al., 2018; He and He, 2020). An example of this is melatonin promoting the production of NO in tomato plants when they were exposed to alkaline stress. In this situation, NO could be a downstream signal that plays an crucial role in the tolerance enhanced by melatonin in tomato plants grown under alkaline stress (Liu et al., 2015). Melatonin, together with NO, promotes plant growth and physiological function. The current review suggests that the mechanism of melatonin’s interaction with NO in plants under abiotic stress is still not clearly understood, and the various genes activated as a result of that interaction have not yet been identified.

### Melatonin regulated the transport and distribution of auxin

**Figure 4:**
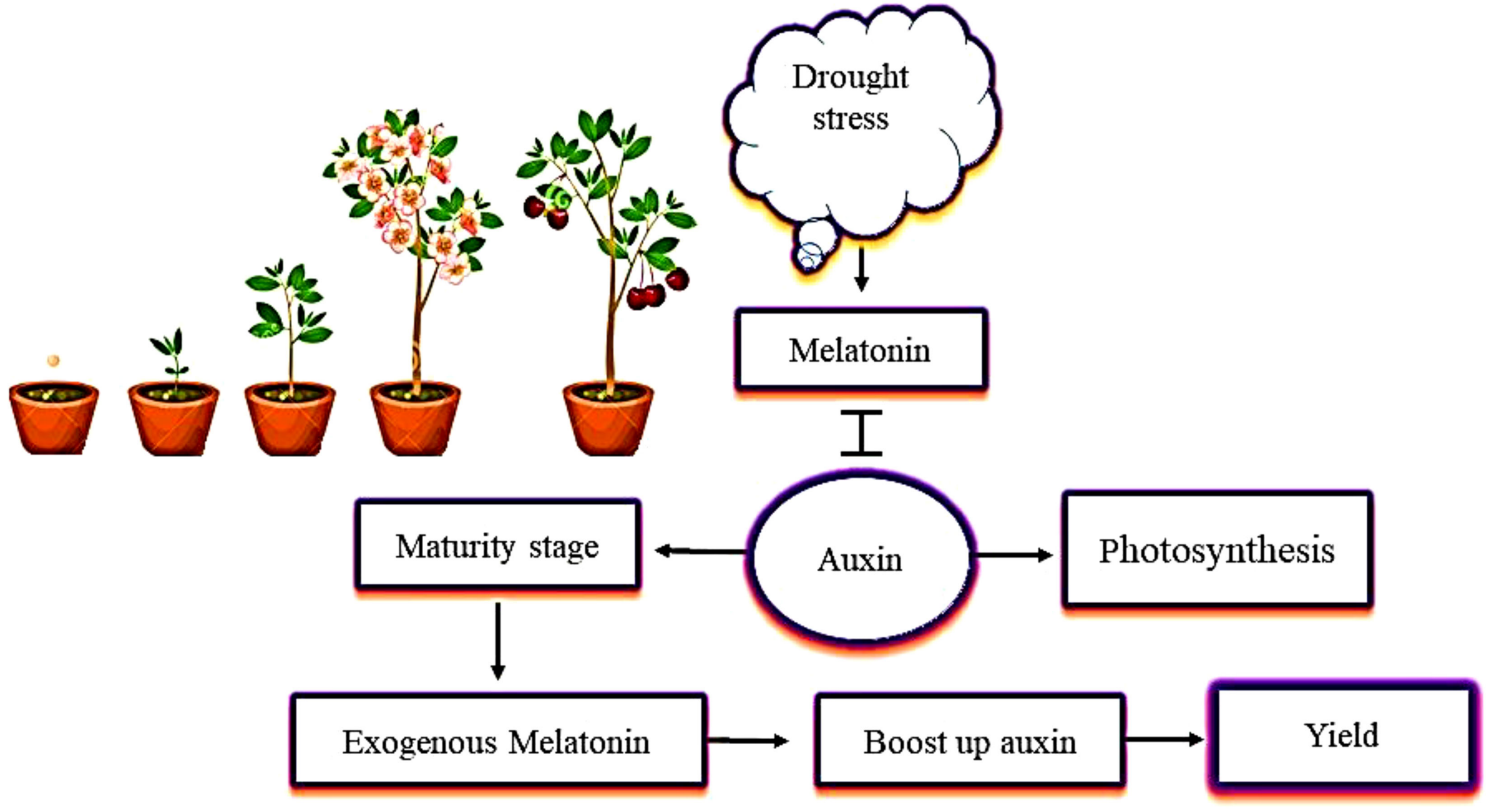
The interactive role of melatonin with auxin (IAA, indole-3-acetic acid) in mitigating the effects of drought stress. The application of melatonin increases IAA content in plants. Melatonin and IAA promote plant photosynthetic activity in the same way. When plants are under drought stress, melatonin interacts with IAA at the maturity stage, and increases plant growth and yield.

Previous studies of the relationship between melatonin and auxin have focused on their chemical similarity (Arnao and Hernández-Ruiz, 2021). Melatonin promotes growth by increasing the concentration of IAA, synthesis of IAA, and polar IAA transport (Wang et al., 2016; **Figure 4**). Various studies have also identified the ability of melatonin and auxin to regulate root and shoot growth and to promote photosynthesis in a similar way (Tan et al., 2019; Mao et al., 2020). A study of plants under drought stress showed that melatonin encouraged the plants to produce more IAA, which helped to increase plant growth and yield. During the maturity stage, the concentration of melatonin decreased, and the increase in IAA concentration was negligible (Ahmad et al., 2022). This decrease in IAA concentration seen in plants in the later growth stages is due to the decreased demand for IAA (Jia et al., 2020). Another similar study showed that the content of IAA decreased from the early growth to the maturity stages in plants under drought stress. Thus, it is conceivable that plants need higher levels of IAA during seedling growth. Plants require certain hormones during their growth and development. Melatonin boosts the IAA levels in plants, and IAA plays an indispensable role in the growth of plants and their development from germination to maturity.

## Role of melatonin in plants under abiotic stress

### Salinity

Publishedexamples of melatonin mitigating abiotic stresses in various species of cropplants are shown in **Table 1**. Salt stress decreased chlorophyll content and photosynthetic activity and enhanced ROS activity and photoperiod regulation (Yin et al., 2019). Melatonin improved thegrowth of green bean seedlings under salt stress, increased photosynthetic activity, and mitigated the oxidative damage caused by ROS by improving antioxidant defense systems in plants (Hasanuzzaman et al., 2020; Elsayed et al., 2021). Multiple studies indicate that melatonin plays a vital role in adaptive responses to salt stress in various plant species (Chen et al., 2018; Liang et al., 2018). However, most of these studies are observational and the findings have not been supported by physiological and molecular research (Liu et al., 2020). In rice, melatonin enhanced salt stress by enabling K^+^ retention (a vital component of plant tissue tolerance mechanisms) in the roots of plants, and by enabling the process that required Oryza sativa (OS) respiratory burstoxidase homolog F (OsRBOHF)-dependent ROS signaling to trigger stress-responsive genes, which in turn increased the expression of K^+^ uptake transporters (particularly *OsHAK5*) in the tips of roots *(* Liu et al., 2020 *)*. *Potassium is an essential element for plant growth and development, and its reduction has been observed under salt stress* (Chen et al., 2018; Liu et al., 2019). These results correspond with the findings of Huang et al. (2019), who reported that NaCl-induced respiratory burst oxidase homolog (RBOH)-mediated production of H_2_O_2_ may be essential for stress signaling and plant adaptation to saline stress. However, studies on the role of OsRBOHF-dependent ROS signaling in the activation of stress-responsive genes and increased expression of K^+^ uptake transporters *in the root tip of plants are lacking, as they have not been conducted on a large variety of plants under abiotic stresses. In addition, further research should focus on identifying responsive genes from OsRBOHF-dependent ROS signaling to increase the uptake of K^+^ transporter ions in the root tips of different crops under various stresses* (Yu et al., 2018).

**Table 1:** Published examples of melatonin in mitigating abiotic stress in various crops.

Abiotic stress	Crop	Response of plants treated with melatonin under abiotic stress	References
Salt stress	Rice	Increased salt tolerance	Liu et al., 2020
	Tobacco	Increased salt tolerance	Cao et al., 2006
	Melon	Increased salt tolerance	Castañares and Bouzo, 2019
	*Limonium bicolor*	Increased salt tolerance	Li et al., 2019
	*Grapevine*	Increased salt tolerance	Xu et al., 2019
Drought stress	Corn	Increased drought tolerance	Li et al., 2021
	Apple	Increased drought tolerance	Li et al., 2015
Heat stress	*Arabidopsis*	Increased heat tolerance	Hernández et al., 2015
	Tomato	Increased heat tolerance	Wang et al., 2018
Cold stress	Corn	Increased cold stress tolerance	Posmyk et al., 2009b
	Cucumber	Increased cold stress tolerance	Kolodziejczyk et al., 2016
	*Arabidopsis*	Increased cold stress tolerance	Shi et al., 2015b
	Bermuda grass	Increased cold stress tolerance	Khalid et al., 2022
Heavy metal stress	Wheat	Increased heavy metal tolerance	Zeng et al., 2022
	Tomato	Increased heavy metal tolerance	Hasan et al., 2019
	*Arabidopsis*	Increased heavy metal tolerance	Yin et al., 2022
	Rice	Increased heavy metal tolerance	Maharajan et al., 2022
Other stresses	*Malus hupehensis*	Increased UV stress tolerance	Wei et al., 2019
	Mediterranean	Increased UV stress tolerance	Nawaz et al., 2022
	Alpine species	Increased UV stress tolerance	Nawaz et al., 2022

UV, ultraviolet light.

Melatonin also promotes ethylene biosynthesis, and the application of melatonin was found to strongly induce *MYB108A* and *ACS1* genes during grape berry ripening (Xu et al., 2017). The *MYB108A* and *ACS1* genes, which perform their function as transcription and essential genes that participate in the production of ethylene, were induced by the application of melatonin (Dong et al., 2011). *ACS* genes are considered a significant target under abiotic stresses to regulate ethylene production in plants. The salt-responsive gene *VviACS1* has been identified as being responsible for ethylene production in plants (Xu et al., 2019). In addition, the *ACSa* and *ACS1* genes are significant in that they are considered a primary target for salt tolerance in corn and tobacco (Cao et al., 2006; Lee and Back, 2016). *Melatonin, combined with 1-aminocyclopropane-1-carboxylic acid (ACC, an ethylene precursor), improved salt tolerance in grapevine plants. In addition, ethylene production was involved in melatonin-induced salt tolerance (* Xu et al., 2019 *). The mechanism and function of MYB108A, ACS1, ACSa*, and *VviACS1* genes in ethylene production due to the melatonin induction under abiotic stresses in different plants is largely unknown.

### Drought

Drought stress negatively affects plants’ morphophysiological and biochemical activity, leading to a decrease in crop yields (Singh et al., 2015; Chen et al., 2019). Drought stress is the cause of oxidative stress and damages plant cells, and, *via* the higher accumulation of ROS, decreases stomatal closure and photosynthetic activity, and results in a deterioration of antioxidant defense systems. The accumulation of ROS is considered a threat to the survival of plant cells as it leads to electron leakage, lipid peroxidation, and subsequent membrane injury, as well as damaged protein and nucleic acid contents (Maksup et al., 2014). To prevent this damage, plants have developed various strategies to regulate their growth under different environmental stresses (Kim and Kim, 2020). As a new plant growth regulator, melatonin is thought to be involved in drought stress responses (Zhang et al., 2015; Li et al., 2021). Drought stress reduced morphological activity in plants, including that pertaining to leaf size and the relative water conductivity of corn seedlings. Meanwhile, both leaf size and relative water conductivity were significantly enhanced by the application of melatonin (Li et al., 2021). A similar result was revealed by Ye et al. (2016), who reported that melatonin improved the shoot dry weight and leaf size of corn seedlings. In plants, physiological processes in leaves, such as photosynthesis, respiration, and transpiration, are maintained by stomata, the opening and closing of which are controlled by complex signal transduction pathways and water balance. In the presence of drought stress, plants regulate their cellular moisture content by regulating stomatal closure and reducing their transpiration rate. However, the density of stomata significantly increases with the contraction of guard cells, and deteriorates under drought stress (Xue et al., 2021). In general, the application of melatonin has shown resistance against the deterioration of stomata cells and increased its length and width under drought stress in corn (Li et al., 2021). The contrasting results in the study by Li et al. (2015), however, demonstrated that drought stress did not reduce stomatal cell density in apples. Nevertheless, the exogenous supply of melatonin maintained high turgor pressure and kept the stomata open. The difference in the findings might be because of the differences in the regulatory mechanism of melatonin in different plant species (Li et al., 2021). The present review demonstrates that melatonin’s quantity, performance, and mechanisms of action differ from plant species to plant species, but fewer morphophysiological responses have been documented under drought stress in different plants.

### Heat stress

High levels of heat stress increase endogenous melatonin concentrations and, thereby, enhance thermotolerance, because of the potent antioxidant capacity of melatonin in plants (Liang et al., 2018; Ahammed et al., 2019). A previous study on *Arabidopsis* plants demonstrated that melatonin increased the seed germination rate from approximately 30% to 39% under heat stress (Hernández et al., 2015). It has been confirmed by the correlation between the synthesis of phytomelatonin and seed germination that phytomelatonin is synthesized during the germination of cucumber seeds, and that its synthesis peaks 14 hours after germination (Zhang et al., 2014). Nevertheless, further research on various crops is still needed. Melatonin improved germination capability by promoting soluble sugar utilization and synthesis of new proteins, and increased amylase and α-amylase activities in melon and *Limonium bicolor* seeds (Castañares and Bouzo, 2019; Li et al., 2019). Recent research has revealed the mechanisms by which melatonin significantly mitigates the effects of heat stress on plant seeds. First, because of the high potency of melatonin, it maintained high viability and germination capacity (Hernández et al., 2015). When plants are exposed to high levels of heat stress, the activities of antioxidant enzymes, such as superoxide dismutase (SOD), peroxidase (POD), and catalase (CAT), are increased (Wang et al., 2022), and melatonin inhibits the accumulation of H_2_O_2_ (Marta et al., 2016). Melatonin treatment up-regulates genes, such as GA20ox and GA3ox, which are involved in gibberellin (GA) biosynthesis. The content of GA, particularly GA_4_, is also increased by melatonin. However, unfortunately, melatonin down-regulates the expression of the essential gene *NECD2*, which is mainly involved in ABA biosynthesis (Zhang et al., 2014; Li et al., 2019). The mechanisms of the up-regulation and down-regulation of gene expression should be studied further in plant cells under heat stress.

Furthermore, heat stress can deteriorate the balance between antioxidants, resulting in ROS accumulation and causing peroxidative damage to cell membranes (Sun et al., 2021). The exogenous application of melatonin in tomatoes and rice reversed the adverse effects of heat stress on plant shoot and root growth (Wang et al., 2018). Melatonin also reduced the damage caused by heat stress by regulating redox homeostasis, and modulating NO and polyamine biosynthesis in tomato seedlings (Jahan et al., 2019). In *Arabidopsis* plants, the heat shock protein HSP_90_ and heat shock factors (i.e., HSFA_2_ and HSFA_32_) contributed to the alleviation of melatonin-mediated heat stress (Shi et al., 2015a). A study demonstrated that HSPs prevented the cellular proteins of tomatoes, grown under heat stress, from refolding or degrading denatured proteins (Xu et al., 2016). Heat shock proteins (HSPs) can isolate and store unfolded proteins. In addition, HSPs can act as chaperones by protecting cells against stresses that can induce protein denaturation and block protein aggregation, and by enhancing the survival of cells and, in turn, of the cellular activity during high levels of heat stress. However, our current understanding of how heat shock proteins and heat shock factors relate to melatonin-mediated heat stress is limited, and in need of further investigation.

### Cold stress

Cold stress is one of the major abiotic stresses that reduces crop growth and yield, especially in temperate zones and highly elevated areas (Bhat et al., 2022). Plants exposed to cold stress experience changes in various physiological, molecular, metabolic, and biochemical activities. Examples include variations in membrane fluidity, metabolism homeostasis, and enzyme activity (Wu et al., 2022). Photosynthesis is a pivotal plant metabolism process, and one that is highly sensitive to cold stress. This is because low temperature hinders many major components of photosynthesis (Dahal et al., 2012). Chlorophyll content decreases under cold stress, leading to chlorosis in leaves (Kaura et al., 2022). The chlorophyll content of leaves provides important information about the effectiveness of physiological processes in plants in plants (Gitelson et al., 2003). Plants treated with melatonin had a higher concentration of chlorophyll than non-treated plants under cold stress (Yang et al., 2022). Plant growth at low temperatures induces the excessive production or inefficient deactivation of ROS, such as H_2_O_2_, superoxide anions (i.e., O_2_^–^), and hydroxyl radicals (i.e., OH), which in turn can cause injury to plants (Ghaderian et al., 2015). In addition, ROS accumulation causes the oxidation of proteins and peroxidation of lipids within plant cells, resulting in reduced plant growth (Nahar et al., 2015). For self-defense against oxidative injury caused by ROS, plants have evolved effective antioxidant systems to scavenge ROS, such as SOD, POD, and CAT, as well as non-enzymatic antioxidants, including proline and glutathione (Erdal et al., 2015; Ghaderian et al., 2015). Several studies have demonstrated that exogenous melatonin can stimulate plant growth in various plants, such as corn, and can promote the germination of cucumber seeds under cold stress (Posmyk et al., 2009b; Kolodziejczyk et al., 2016). In *Arabidopsis* plants, melatonin modulates leaf senescence against cold stress (Shi et al., 2015b). Melatonin applications enhance the resistance of Bermuda grass to cold stress by improving cell membrane stability, and by regulating photosynthesis and metabolic activity (Khalid et al., 2022). Melatonin played a role as both a first-line defense and internal sensor of oxidative stress in a study of different species of plants (Iqbal and Khan, 2022). For example, in barley, exogenous melatonin can enhance photosynthetic carbon assimilation by improving the plant antioxidant defense systems of organelles under cold stress (Li et al., 2016). Therefore, the improved performance of primed seeds in terms of seedling growth and germination might be the result of improved antioxidant defense systems under cold stress (Cao et al., 2022). However, an understanding of the growth of waxy corn and other crop seeds primed with melatonin in response to cold stress is still limited (Cao et al., 2022).

### Heavy metal stress

Certain heavy metals, such as zinc (Zn), cadmium (Cd), iron (Fe), and copper (Cu), are essential for plant growth and metabolism, but their accumulation to higher levels can negatively affect plant growth and yield. Heavy metal stress inhibits plant photosynthetic activity, the activity of enzymes involved in the Calvin cycle, and carbohydrate metabolism (Hasan et al., 2019). In addition, higher levels of ROS accumulation during heavy metal stress inhibit root growth and promote leaf senescence in turn, chloroplasts (Zeng et al., 2022). Previous studies have demonstrated that plants treated with melatonin can improve their growth and yield by improving their morphophysiological activities under heavy metal stress conditions. The production of endogenous melatonin in plants can be triggered by the application of exogenous melatonin which builds up heavy metal tolerance (Menhas et al., 2022). Melatonin enhanced plant metabolism and antioxidant enzymes activity, and triggered the ascorbate–glutathione cycle to counteract the effects of heavy metal stress (Moustafa-Farag et al., 2020). In wheat seedlings, exogenous melatonin increased endogenous melatonin and, as a result, enhanced root and shoot growth under cadmium (Cd) toxicity (Zeng et al., 2022). The increase of endogenous melatonin mitigates cadmium toxicity by balancing H_2_O_2_ homeostasis and activating antioxidant defense systems in wheat (Ni et al., 2018). Melatonin effectively mitigated Cd toxicity by improving H^+^-ATPase activity and phytochelatin and glutathione content, and by facilitating Cd sequestration in tomato plant cells (Hasan et al., 2015). Melatonin impacted sulfur metabolism, which plays an important role in plant tolerance against Cd stress (Menhas et al., 2022). In tomatoes, melatonin deficiency reduced the sulfur concentration and increased the accumulation of cadmium (Hasan et al., 2019). The overexpression of melatonin biosynthetic enzymes genes improved heavy metal stress in *Arabidopsis* plants (Yin et al., 2022). Similarly, in rice, various gene families, such as *NRAMP*, *HMA*, *MTP*, *YSL*, and *ZIP*, are involved in heavy metal stress (Maharajan et al., 2022). These genes reduced the uptake of heavy metals and accumulation in rice grains (Peris-Peris et al., 2017). Several studies investigating the role of melatonin in plant morphophysiological activity, antioxidant capacity, and biosynthetic genes in various crops have recently been undertaken. However, the role of melatonin in improving these activities, as well as the transduction pathways of different genes in cotton, rice, and other crops under heavy metal stress, is still unknown.

### Other stresses

Ultraviolet (UV) radiations negatively effects plant growth and development, andtheir intensity continuously increasing caused by rapid ozone layer depletion. The higher levels of UV radiation can substantially reduce crop productivity by hindering plant PSII, photosynthetic activity, nucleic acids, and biomass accumulation and portioning (Bera et al., 2022). Plants exposed to higher levels of UV radiation have reduced expression and synthesis of key photosynthetic proteins, such as chlorophyll *a/b* binding proteins (Khudyakova et al., 2019). Melatonin plays a vital role in mitigating the negative effects of UV radiation on crop productivity. It has been demonstrated that exogenous melatonin in *Malus hupehensis* and *Nicotiana sylvestris* plants facilitates the UV-induced damage to DNA and UV radiation induced by ROS (Wei et al., 2019). Melatonin is considered a potent antioxidant that protects plants against UV radiation; it regulates the expression of various UV signaling pathways, such as transcription factors RUP1/2, HY5, and HYH, and the ubiquitin-degrading enzyme COP1 (Yao et al., 2021). Exogenous melatonin improved the expression of RUP1/1, HY5, HYH, and COP1, which perform a key role in the protection against UV radiation (Hassan et al., 2022). Hence, melatonin regulates antioxidant defense systems to prevent plants from the negative impacts of UV stress (Yao et al., 2021). Endogenous melatonin is substantially increased in *Glycyrrhiza uralensis* plants when their roots are exposed to UV radiation, and as a result plant DNA damage is reduced (Wei et al., 2019). Similarly, the accumulation of endogenous melatonin induces a tolerance response to UV stress in Mediterranean and alpine species of plants (Nawaz et al., 2022). Although limited research has demonstrated a role for melatonin in UV stress tolerance (Hassan et al., 2022), more studies are required to investigate the role of melatonin in regulating various UV signaling pathways that are involved in mitigating the negative effects of UV radiation in various crops.

## Conclusions and future directions

The impact of abiotic stresses on plant development is considered a significant threat to agricultural productivity. Plants adopt different physiological, biochemical, and molecular responses to overcome the negative effects of abiotic stresses (Ahmad et al., 2022b). Phytomelatonin has potential to be used as a tool for reducing or alleviating the adverse effects of abiotic stresses in various crops. The exogenous application of melatonin is essential for plant growth and development under abiotic stresses. Phytomelatonin plays a key role in plant metabolism and the complex mechanism of plant function; however, the role that melatonin plays in the underlying mechanisms in plants grown under abiotic stress is still poorly understood. Moreover, the interaction of melatonin with NO and with IAA/auxin, and their responses to abiotic stresses, make for attractive targets in molecular research. The relationship between melatonin and NO regulates morphophysiological and biochemical activities by way of the G protein-coupled receptor and synthesis genes. Furthermore, the mechanism by which G protein regulates the morphophysiological activity and the different genes involved in the regulation by melatonin are still unclear. In addition, the interaction of melatonin with auxin enhanced growth and physiological function by increasing the levels of auxin, synthesis, and polar transport. In the later growth stage, the content of auxin is decreased because of the decreased melatonin concentrations in plants. To grow and achieve a higher yield, plants need a continuous supply of IAA from sowing to maturity. In the lateral growth stage, the effects of an exogenous supply of melatonin, and the mechanism by which melatonin boosts IAA levels in various crops, are still unknown.

Melatonin has an important role in regulating plant metabolism and increasing yield under various abiotic stresses. In addition, the OsRBOHF-dependent ROS signaling that activated stress-responsive genes in plants grown under abiotic stress enhanced the uptake of potassium (K^+^) transporter (*OsHAK5*) in the roots. The potassium transporter *OsHAK5* plays a vital role in potassium acquisition and transport from root tissue to the shoots, especially in plants exposed to low potassium concentrations, enhancing plant metabolism and physiological function under salt stress. The mechanism of the K^+^ transporter *OsHAK5* and the activation of gene identification, which are due to the OsRBOHF-dependent ROS signaling in various crops under abiotic stresses, however, is poorly understood.

Ethylene in plants is considered a multifunctional phytohormone that significantly improves plant growth and senescence. However, the role of genes such as *MYB108A*, *ACS1*, *ACSa*, and *VviACS1* in ethylene production in different plants under various stresses is still poorly understood.

In addition, serotonin plays a vital function in plants, acting as a growth regulator and as a stress defense molecule. The relationship of melatonin with *T5H* genes in serotonin biosynthesis under abiotic stress is still unknown in many plants.

Stomata density is closely associated with plant growth properties, and photosynthetic activity is improved by melatonin application. Stomatal cells undergo deterioration when plants are exposed to drought stress (Salehi-Lisar and Bakhshayeshan-Agdam, 2016). In contrast, no adverse effects on stomatal density were observed in apple plants grown under drought stress (Li et al., 2015). The reason for these different results might be that there are different signaling pathways in different crops. The mechanisms of this phenomenon in various crops are still poorly understood. These mechanisms need to be further investigated under various stresses because the amount performance, and mechanism of action of melatonin vary among plant species.

It has been confirmed that, melatonin up-regulates *GA20ox* and *GA3ox*, genes that are involved in GA biosynthesis and result in increased GA_4_ while down-regulating the *NECD2*, which is involved in ABA biosynthesis. The role of melatonin in the up- and down-regulation of genes involved in the biosynthesis of GA and ABA under various abiotic stresses remains unclear.

The heat shock proteins of tomatoes protect the plants’ cellular protein against heat stress due to refolding or degradation of denatured proteins. However, the response and activity of heat shock proteins and heat factors in plants with a melatonin supply under abiotic stresses in different crops is still poorly understood.

## Author contributions

IA was involved in the conceptualization, writing/reviewing, and editing the original draft. GuiZ was involved in supervision. MI and GuaZ were involved in collection of the literature. YJ and XS contributed to the writing of the manuscript. AA and MY eliminated grammatical errors. All authors contributed to the article and approved the submitted version.
